# Phase I clinical trial of autologous NK cell therapy using novel expansion method in patients with advanced digestive cancer

**DOI:** 10.1186/s12967-015-0632-8

**Published:** 2015-08-25

**Authors:** Naoyuki Sakamoto, Takeshi Ishikawa, Satoshi Kokura, Tetsuya Okayama, Kaname Oka, Mitsuko Ideno, Fumiyo Sakai, Akiko Kato, Masashige Tanabe, Tatsuji Enoki, Junichi Mineno, Yuji Naito, Yoshito Itoh, Toshikazu Yoshikawa

**Affiliations:** Department of Molecular Gastroenterology and Hepatology, Graduate School of Medical Science, Kyoto Prefectural University of Medicine, Kyoto, Japan; Department of Cancer ImmunoCell Regulation, Graduate School of Medical Science, Kyoto Prefectural University of Medicine, Kyoto, 602-8566 Japan; Iseikai Hyakumanben Clinic, Kyoto, Japan; Center for Education Research and Development, Kyoto Gakuen University, Kyoto, Japan; CDM Center, Takara Bio Inc, Otsu, Japan

## Abstract

**Background:**

NK cells can destroy tumor cells without prior sensitization or immunization. Tumors often lose expression of MHC molecules and/or antigens. However, NK cells can lyse tumor cells in a non-MHC-restricted manner and independent of the expression of tumor-associated antigens. NK cells are therefore considered ideal for adoptive cancer immunotherapy; however the difficulty of obtaining large numbers of fully functional NK cells that are safe to administer deters its clinical use. This phase I clinical trial seeks to address this obstacle by first developing a novel system that expands large numbers of highly activated clinical grade NK cells, and second, determining if these cells are safe in a mono-treatment so they can be combined with other reagents in the next round of clinical trials.

**Methods:**

Patients with unresectable, locally advanced and/or metastatic digestive cancer who did not succeed with standard therapy were enrolled. NK cells were expanded ex vivo by stimulating PBMCs with OK432, IL-2, and modified FN-CH296 induced T cells. Patients were administered autologous natural killer cell three times weekly via intravenous infusions in a dose-escalating manner (dose 0.5 × 10^9^, 1.0 × 10^9^, 2.0 × 10^9^ cells/injection, three patients/one cohort).

**Results:**

Total cell population had a median expansion of 586-fold (range 95–1102), with a significantly pure (90.96 %) NK cell population. Consequently, NK cells were expanded to approximately 4720-fold (range 1372–14,116) with cells being highly lytic in vitro and strongly expressing functional markers such as NKG2D and CD16. This NK cell therapy was very well tolerated with no severe adverse events. Although no clinical responses were observed, cytotoxicity of peripheral blood was elevated approximately twofolds up to 4 weeks post the last transfer.

**Conclusion:**

We successfully generated large numbers of activated NK cells from small quantities of blood without prior purification of the cells. We also determined that the expanded cells were safe to administer in a monotherapy and are suitable for the next round of clinical trials where their efficacy will be tested combined with other reagents.

Trial Registration: UMIN UMIN000007527

**Electronic supplementary material:**

The online version of this article (doi:10.1186/s12967-015-0632-8) contains supplementary material, which is available to authorized users.

## Background

Natural killer (NK) cells play critical roles in the early innate response to pathogens and tumor cells [1, 2]. These cells exhibit strong cytotoxic activity against tumor cells without prior sensitization or immunization, and produce numerous cytokines resulting in the subsequent activation of the adoptive immune system.

Tumors often lose expression of tumor-associated antigens and/or MHC molecules as a means of immune escaping detection by T cells [3–5]. NK cells can lyse tumor cells in a non-MHC-restricted manner and are independent of the expression of tumor-associated antigens. Due to this, NK cells are considered ideal for adoptive cancer immunotherapy. In contrast to vaccine therapy or antigen-specific adoptive T cell therapy, it is not necessary to identify target tumor antigen for NK cell-based immunotherapy; this makes it more universally applicable and particularly effective for treating solid tumors that frequently lose tumor-associated antigens and/or self-MHC molecules. NK cell-based immunotherapy has been recommended as a means to improving hematologic malignancies [6, 7] and solid tumors [8–12] in clinical settings.

NK cells seem to possess many advantages that would make it ideal for clinical application. However, existing drawbacks are that it is difficult to generate large numbers of fully functional NK cells, and a standard method of ex vivo NK cell expansion has not been established yet. T cells can be expanded more than 1000-fold ex vivo using anti-CD3 monoclonal antibody in combination with cytokines and other stimuli [13, 14]. However in general, NK cells cannot sustain proliferation, therefore, their proliferative responses to cytokines with or without being co-cultured with other cells is modest and temporary [15–17]. To overcome this obstacle, researchers are seeking to develop new methods to obtain larger populations of highly pure NK cells. Examples include the ex vivo expansion of NK cells from (1) leukapheresis products by immunomagnetic beads selection [18–20], (2) from hematopoietic stem and progenitor cells from umbilical cord blood [21, 22], and (3) cytokine-based expansion method co-cultured with transgenic or irradiated tumor cells, and irradiated peripheral blood mononuclear cells [23, 24]. While these methods [18–24] have some merit, they have major drawbacks including: low expansion scale [20], low purity of NK cells [24], high cost [18–20], complicated procedures [18–24], and safety issues for human administration [23]. Developing innovative strategies to generate clinically relevant pure NK cells in large numbers would provide an important breakthrough in NK cell-based immunotherapy.

With this in mind, we recently developed a novel clinical-grade NK cell expansion system using recombinant human fibronectin fragment (FN-CH296, RetroNectin^®^)-induced T-cells (RN-T cells) as a stimulator. This method delivered a 688 ± 76-fold expansion of total cells in a sample of 31 cancer patients with purity levels of 84.7 ± 3.6 % without prior purification (Additional file 1: Table S1) [25]. Moreover, the majority of expanded cells highly expressed functional markers such as NKG2D (97.3 ± 0.6 %) and CD16 (96.8 ± 0.7 %) and exerted strong cytotoxicity in vitro and in experimental models of human tumors [25]. Having successfully produced these high quality NK cells, our secondary goal was to evaluate their safety when administered to patients with advanced digestive cancers and also assessed their efficacy as a minor objective.

The maximum tolerated dose for NK cells has not yet been established and no general range has been suggested. In fact, there is conflicting data surrounding the maximum tolerated dose. In prior research the administration of a large number of NK cells (almost 5–25 × 10^9^ cells for 50 kg patient) in conjunction with lymph depleting chemotherapy and systemic IL-2 administration was found to be effective and safe [6–8]. On the other hand, severe adverse events have been reported when fewer (0.4–2.0 × 10^9^ cells for 50 kg patient) NK cells were administered [8]. Taking these conflicting results into consideration, in this phase I clinical study, we evaluated the safety of autologous NK cells generated by our novel system by administered advanced digestive cancer patients with 0.5–2.0 × 10^9^ cells (a similar dose to that used in prior studies [6–9, 11, 12]) a total of three times.

## Methods

### Eligibility

Patients with un-resectable, locally advanced and/or metastatic digestive cancer that was histologically confirmed were enrolled in this study. All patients had failed prior standard therapy and were recruited between September 2012 and June 2013. Eligibility criteria included: age >20 years, Eastern Cooperative Oncology Group (ECOG) performance status ≤2, no plans to receive chemotherapy other than oral fluorouracil prodrugs or radiation therapy, a life expectancy of at least 3 months, absence of serious cardiovascular disease, adequate vital organ function as indicated by leukocyte count ≥3000/mm^3^, neutrophil count ≥1500/mm^3^, platelet count ≥100,000/mm^3^, hemoglobin ≥9.0 g/dL, serum aspartate aminotransferase (AST) and alanine aminotransferase (ALT) ≤100 IU/L, serum total bilirubin ≤2 mg/dL, serum creatinine ≤1.5 mg/dL, and blood urea nitrogen level ≤25 mg/dL. Patients were excluded if they tested positive for hepatitis B or C virus, HIV, HTLV-1, syphilis infection, had active severe infection, serious complications such as severe diabetes mellitus, unstable angina or myocardial infarction within 3 months, were pregnant or lactating, had a medical history of severe hypersensitivity or autoimmune disease.

### Study design

This was a non-randomized, open label, phase I clinical trial with dose escalation of NK cells. The primary end-point of this study was to assess the safety of the NK cells derived from our method, and the secondary end-points were clinical and immunological responses. This study was approved by the ethics committee of Kyoto Prefectural University of Medicine. The trial was designed and conducted in accordance with the Helsinki Declaration and the Ethical Guidelines for Clinical Research (the Ministry of Health, Labor and Welfare, Japan). All participants provided written informed consent. This trial was registered as the University Hospital Medical Information Network (UMIN) Clinical Trial Registry as ID: UMIN000007527.

### Cell processing

Preparation of the FN-CH296 (RetroNectin^®^)-stimulated T (RN-T) cells.

Peripheral blood (10–20 mL) was taken from each cancer patient. FN-CH296 (RetroNectin^®^, Takara Bio, Shiga, Japan)-stimulated T (RN-T) cells were prepared by a previously described method [13]. Briefly, peripheral blood mononuclear cells (PBMCs) were separated using Ficoll-Paque PREMIUM (GE Healthcare, Tokyo, Japan). Subsequently, 2 × 10^6^ of cells were re-suspended in GT-T551 culture medium (Takara Bio) containing heat-inactivated autologous plasma (0.5 %) and recombinant IL-2 (Proleukin; NovartisPharma, Nürnberg, Germany), then transferred to a cell-culture immobilized with both anti-CD3 mAb (OKT3: Janssen Pharmaceutical k.k, Tokyo, Japan) and FN-CH296. On day 4, the cells were transferred to CultiLife^®^ 215 bag (Takara Bio), and diluted with GT-T551 medium containing plasma (0.5 %) and IL-2 every 3 or 4 days. Cells were cultured for 1–2 weeks, γ-irradiated and then used as stimulators for NK-cell expansion.

#### Large-scale expansion of NK cells

PBMCs were obtained from the peripheral blood (20–40 mL) of each cancer patient. Subsequently, 5.6 × 10^6^ of PBMCs were re-suspended in GT-T507α culture medium supplemented with heat-inactivated autologous plasma (1.0 %), IL-2 and OK-432 (Picibanil: Chugai Pharmaceutical Co, Tokyo, Japan). Cells were then transferred to a cell-culture flask and RN-T cells as a stimulator were added. On day 7, the cultured cells were transferred to a CultiLife^®^ 215 bag and stimulated again by RN-T cells in GT-T510 culture medium (Takara Bio) supplemented with heat-inactivated autologous plasma (1.0 %) and IL-2. On day 11, the cells were transferred to a CultiLife^®^ Eva bag (Takara Bio), and GT-T510 containing plasma (1.0 %) and IL-2 were added. Cells were then expanded by adding GT-T510 medium and increasing the number of CultiLife^®^ Eva bag as necessary. On days 21 and 22, the cultured cells were harvested, washed and re-suspended in 100 mL of a saline based-solution supplemented with 1 % of human serum albumin (Albuminar; CSL Behring, PA, USA) then administered to patients immediately.

Quality control testing was conducted by assessing samples taken during the culture period and the final product for sterility by the BacT/ALERT (bioMérieux, Durham, NC, USA) microbiological detection system and for mycoplasma contamination by a MycoAlert Mycoplasma Detection Kit (Lonza Japan, Tokyo, Japan). Sterility tests were contracted to FALCO Biosystems (Kyoto, Japan). The viability of expanded cells was measured by trypan blue exclusion assay, and tested for endotoxin by a kinetic colorimetric LAL assay. After thawing, a small aliquot of the final product was used for in vitro cytotoxicity assay; it was cryo-preserved and examined to identify the proportions of CD3^−^CD56^+^ cells and other cell surface markers by flow cytometry.

### Treatment protocol

**Fig. 1:**
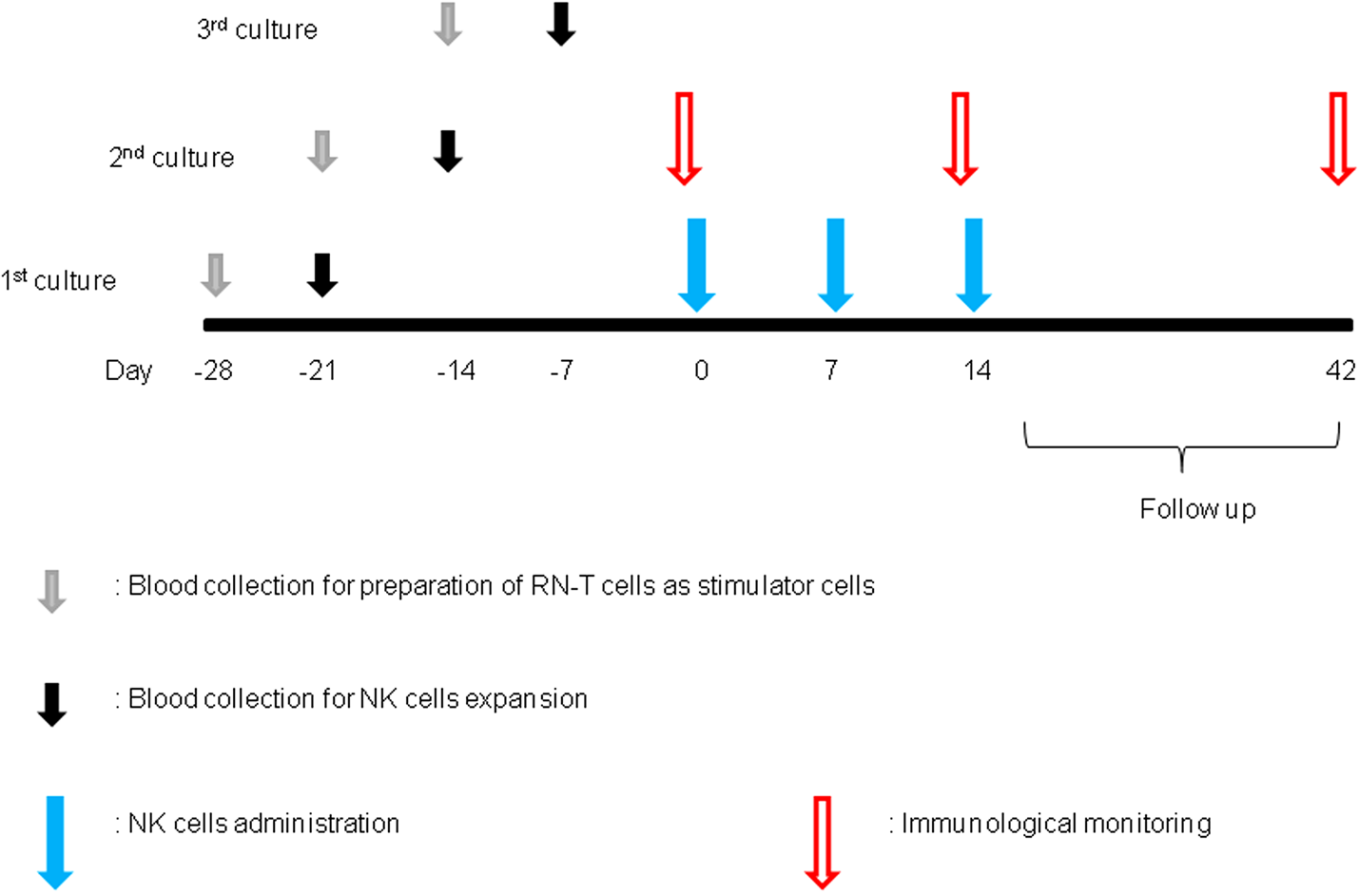
Treatment protocol. PBMCs were separated to prepare RN-T as stimulator cells. One week later, PBMCs were again separated similarly, and re-suspended in culture medium supplemented with heat-inactivated autologous plasma, IL-2 and OK-432. RN-T cells were added to the same flask or culture bag on day 0 and day 7. On days 21–22, the cultured cells were harvested and administered to the patients immediately. Expanded NK cells were intravenously injected for 60 min on days 0, 7, 14 in a dose-escalating manner (dose 0.5 × 10^9^, 1 × 10^9^, 2 × 10^9^ cells/injection, three patients/one cohort). We investigated the dose-limiting toxicity (DLT) occurring over a 28-day period after the last administration of cultured cells. Blood samplings for immune monitoring were done just before the 1st and 3rd administration and 4 weeks after the 3rd administration. *PBMCs* peripheral blood mononuclear cells, *RN-T cells* RetroNectin^®^-induced T cells

The eligibility criteria for transferred cells was as follows: ① The cultured cell viability was more than 80 %. ② The mean purity value of the three cultured cells was more than 50 %. ③ The number of transferred cells was 70–130 % of the number that was set in each cohort. The patients were divided into three cohorts of three to four patients each: Cohort 1, 0.5 × 10^9^ cells per dose; Cohort 2, 1.0 × 10^9^ cells per dose; and Cohort 3, 2.0 × 10^9^ cells per dose. Expanded NK cells that passed quality tests were intravenously injected for 60 min on days 0, 7, 14 (Fig. 1). We investigated the dose-limiting toxicity (DLT) occurring over a 28-day period after the third cell infusion. DLT was defined as grade ≥3 for any adverse event related to the administration of cultured cells. If no DLT was observed in the previous cohort, another cohort was treated at the next higher dose. There was no intra-patient dose escalation in this study.

### Phenotypic analysis

The phenotype of expanded cells and PBMCs at baseline (day 0), before the 3rd administration (day 14), and 4 weeks after 3rd administration (day 42) were analyzed by flow cytometry. Monoclonal antibodies specific for CD3, CD16, CD56 (Beckman Coulter, CA, USA), NKG2D (eBioscience, CA, USA), CXCR3 (R&D system, MN, USA), CXCR4 (Becton–Dickinson, CA, USA), CX3CR1 (Bio Legend, CA, USA) were applied. Each monoclonal antibody was conjugated as follows: CD3 CD16, CD69, CXCR3 with fluorescein isothiocyanate (FITC)-, CD56, NKG2D, CXCR4, CX3CR1 with phycoerythrin (PE)-, CD3 with phycoerythrin-Cyanin 5 (PE-Cy5)-, CD56 with phycoerythrin-Cyanin 7 (PC7-Cy7)-. Cells were analyzed by Cytomics FC500 (Beckman Coulter) and data were acquired by the CXP software, version 2.2 (Beckman Coulter) according to the manufacturer’s instruction.

### Whole blood cytokine assay

Blood samples were obtained from patients on day 0 and on day 42 after the 3rd administration. All plasma samples were stored at −80 °C until they were analyzed. Following the manufacturer’s instructions, we used a Bio-Plex multiplex cytokine array system (Bio-Rad Laboratories, CA, USA) with a panel that quantified the following 27 cytokines: IL-1β, IL-1RA, IL-2, IL-4, IL-5, IL-6, IL-7, IL-8, IL-9, IL-10, IL-12 (p70), IL-13, IL-15, IL-17, basic FGF, eotaxin, G-CSF, GM-CSF, IFN-γ, IP-10, MCP-1, MIP-1α, MIP-1β, PDGF-BB, RANTES, TNF-α, VEGF. Data acquisition and analysis were performed with Bio-Plex Manager Software (version 5.0).

### In vitro cytotoxicity assay

Blood samplings for in vitro cytotoxicity assay were done on day 0, before the 3rd administration (day 14) and on 42 days after the 3rd administration (4 weeks). To evaluate the cytotoxicity of expanded NK cells (final products) fresh PBMCs against K-562 cell as target cell was assayed using DELFIA^®^ cell cytotoxicity kit (PerkinElmer, USA, CA) according to the manufacturer’s instructions. Briefly, PBMCs were added to the well in RPMI 1640 medium with 10 % FCS and incubated for 18 h. Target cells were labeled with Europium-DTPA, and then placed in 96-well tissue culture plates then incubated with PBMCs at various effector-to-target (E:T) ratios. After incubating for 4 h at 37 °C under 5 % CO_2_, the release of Europium-DTPA was measured in a time-resolved fluorometer. Cytotoxicity was calculated as follows: % cytotoxicity = 100 × (experimental release − spontaneous release)/(maximum release − spontaneous release). Calcein-AM(Takara Bio) was used to measure cytotoxicity in cryopreserved expanded NK cells instead of Europium-DTPA, and thawed expanded NK cells was mixed with fluorescent-labeled target cells without pre-incubation. Finally, we calculated the values corresponding to the E:T ratio needed to reduce the cytotoxicity of expanded NK cells by 50 % of the maximum lysis value (EC50).

### Clinical toxicity and efficacy assessment

Safety and toxicity were determined based on regular patient interviews, physical examination and laboratory tests. Safety was assessed and reported according to the National Cancer Institute Common Terminology Criteria for Adverse Events (version 4.0). Objective tumor response was assessed by computed tomography scans in accordance with the Response Evaluation Criteria in Solid Tumor (RECIST VERSION 1.1) criteria. Disease assessment was performed at baseline and every 4 weeks after the final treatment.

### Statistical analysis

The Wilcoxon signed-rank test was used to compare paired samples before and after NK cell therapy and Spearman’s rank correlation tested the association between the two. P values of less than 0.05 were considered statistically significant. Statistical analysis was performed using GraphPad Prism 5 for Windows (GraphPad, San Diego, CA, USA).

## Results

### Patient characteristics

Between September 2012 and June 2013, fourteen patients (11 males and 3 females) were enrolled in this trial. Table 1 shows patient demographics and clinical characteristics. The median age was 65.3 years (range 48–78). Seven (50.0 %) patients had colorectal cancer, four (28.6 %) had esophageal cancer, and three (21.4 %) had gastric cancer. Two patients in cohort 1 were removed from the study because the purity (no. 1, mean 30 %) or the number (no. 2) of expanded cells did not meet the minimum criteria (0.35 × 10^9^ cells per dose). Two patients whose PS was 2 (no. 4 from cohort 1 and no. 10 from cohort 2), had disease progression during the NK cells preparation period and therefore received only two administration of NK cells.

**Table 1:** Patient characteristics

Cohort	Case	Age/gender	Diagnosis	Disease stage	ECOG/PS	Prior treatment	Combined treatment (chemotherapy)	NK cell administration
1	1	57/M	Rectal cancer	Recurrent	1	Surgery, chemotherapy	None	Excluded from trial^a^
	2	74/F	Rectal cancer	Recurrent	0	Surgery, chemotherapy	S-1	Excluded from trial^a^
	3	61/M	Esophageal cancer	Metastatic	2	Chemotherapy, radiation therapy	None	Complete
	4	62/M	Gastric cancer	Recurrent	2	Surgery, chemotherapy	S-1	Incomplete (2 times)
	5	72/M	Esophageal cancer	Recurrent	0	Surgery, chemotherapy	None	Complete
	6	73/M	Colon cancer	Recurrent	0	Surgery, chemotherapy	None	Complete
	7	66/M	Gastric cancer	Metastatic	0	Chemotherapy	None	Complete
2	8	62/M	Esophageal cancer	Recurrent	0	Surgery, chemotherapy, radiation therapy	None	Complete
	9	65/M	Colon cancer	Metastatic	0	Chemotherapy	S-1	Complete
	10	67/M	Esophageal cancer	Metastatic	2	Chemotherapy, radiation therapy	None	Incomplete (2 times)
	11	69/M	Gastric cancer	Metastatic	0	Chemotherapy	None	Complete
3	12	60/M	Colon cancer	Recurrent	0	Surgery, chemotherapy	None	Complete
	13	78/F	Rectal cancer	Recurrent	0	Surgery, chemotherapy	None	Complete
	14	48/F	Rectal cancer	Recurrent	0	Surgery, chemotherapy, radiation therapy	None	Complete

*ECOG* Eastern Coorperative Oncology Group, *PS* performance status

^a^Two patients were excluded from trial because the purity (no. 1) or the number (no. 2) of expanded cells didn’t exceed eligible value

### Characteristics of expanded NK cells

**Fig. 2:**
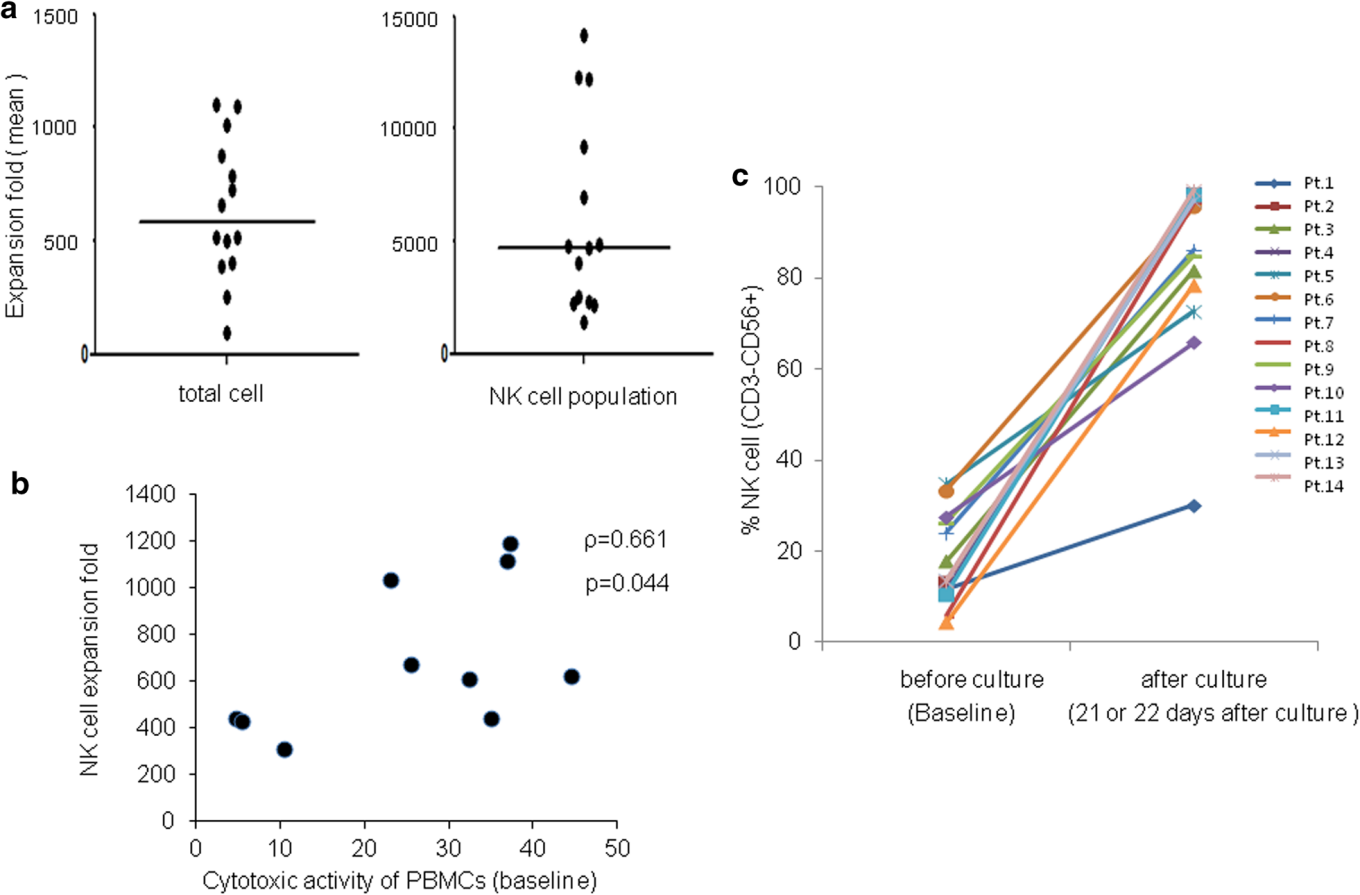
Expansion and NK purity dynamics of PBMCs obtained from 14 patients. **a** Fold expansion of total cell and NK cell population (CD3^−^CD56^+^ cell) during the culture period. *Dots* represent mean values of each patient in triplicate cultures. *Horizontal bars* indicate median values. **b** Relation of NK cell expansion fold and cytotoxic activity of PBMCs. Expansion fold of NK cells in the first culture significantly correlated with cytotoxic activity of PBMCs at baseline (ρ = 0.661, p = 0.044). *PBMCs* peripheral blood mononuclear cells. **c** The purity of NK cell population (CD3^−^CD56^+^ cell) at baseline and 21 or 22 days after the initiation of the culture

PBMCs from 14 enrolled patients were cultured. The median of NK cell (CD3^−^CD56^+^) in lymphocytes was 13.59 % (range 4.43–34.85). The median of total cell or NK cell expansion rate after 21 and 22 days of culture was 586-fold (range 95–1102) and 4720-fold (range 1372–14,116), respectively (Fig. 2a). The total cell expansion fold did not correlate with the percentage of NK cells in lymphocytes (ρ = 0.24, P = 0.40), but in the 1st culture it significantly correlated with the cytotoxicity activity of PBMCs on day 0 (ρ = 0.66, P = 0.04, Fig. 2b). As shown in Fig. 2c, the purity of expanded NK (CD3^−^CD56^+^) cells markedly increased after culture, with the exception of one patient (Pt no. 1). The median purity of NK cell (CD3^−^CD56^+^) was 90.96 % (range 65.94–99.45) in the intention-to treat (ITT) population and 96.14 % (range 65.94–99.45) in the per protocol (PP) population. As shown in Table 2 however, the percentage of CD3^+^CD56^+^, CD3^+^CD4^+^ and CD3^+^CD8^+^ cells was minimal (median 8.60, 3.50 and 0.24 %, respectively). Expanded NK cells highly expressed cell surface markers such as NKG2D and CD16, which are considered viable and functional markers of NK cells. In the ITT population, the median percentage of NKG2D+ and CD16+ cells in NK population was 98.36 % (range 95.20–99.59) and 61.75 % (range 20.84–73.50), respectively. We also found relatively high expression levels of chemokine receptors such as CXCR3, CXCR4 and CX3CR1 on expanded NK cells (median in ITT population, 45.47, 37.71, and 43.74 %, respectively). Additional file 2: Figure S1 shows representative flow cytometry dot-plots for each population of expanded cells in patient no. 14.

**Table 2:** Characteristics of expanded cells

Patient no.	CD3^−^CD56^+^	CD3^+^CD56^+^	CD3^+^CD4^+^	CD3^+^CD8^+^	NKG2D+ in CD3^−^CD56^+^	CD16+ in CD3^−^CD56^+^	CXCR3+ in CD3^−^CD56^+^	CXCR4+ in CD3^−^CD56^+^	CX3CR1+ in CD3^−^CD56^+^	NK activity EC50^a^
1	30.18	62.53	NE	NE	NE	NE	NE	NE	NE	2.61
2	98.41	0.93	0.40	0.24	98.91	23.01	28.33	49.79	36.79	1.31
3	81.78	16.28	17.15	0.96	96.70	20.84	48.80	21.98	28.33	1.31
4	98.75	0.91	0.55	0.13	96.28	29.47	36.67	29.78	28.37	1.61
5	72.84	26.50	25.49	1.87	99.59	68.39	44.82	20.32	39.51	0.63
6	95.67	3.93	3.12	0.30	99.36	60.70	56.62	23.31	42.07	0.62
7	86.25	13.27	3.87	0.24	95.20	50.29	29.04	31.34	26.79	1.39
8	96.60	2.27	2.25	0.07	98.54	60.46	42.90	62.89	50.40	1.24
9	84.91	14.91	10.31	2.64	98.17	72.33	41.80	37.88	62.01	0.98
10	65.94	38.83	33.61	0.23	98.67	62.79	44.04	42.40	51.40	1.88
11	98.49	1.39	0.45	0.11	99.45	69.23	49.69	69.29	44.33	1.76
12	78.52	21.40	11.43	0.30	97.88	73.50	61.05	37.54	70.20	1.99
13	97.23	2.57	2.60	0.11	99.00	32.88	57.66	49.55	54.89	0.59
14	99.45	0.44	0.28	0.09	97.82	63.59	46.12	43.73	43.15	0.39
Median (all enrolled patients)	90.96	8.60	3.12	0.24	98.54	60.70	44.82	37.88	43.15	1.31
Median (ITT population)	90.96	8.60	3.50	0.24	98.36	61.75	45.47	37.71	43.74	1.28

^a^The data are presented for EC50, which means the values corresponding to the E:T ratio needed to reduce the cytotoxicity by 50 % from maximum lysis

**Fig. 3:**
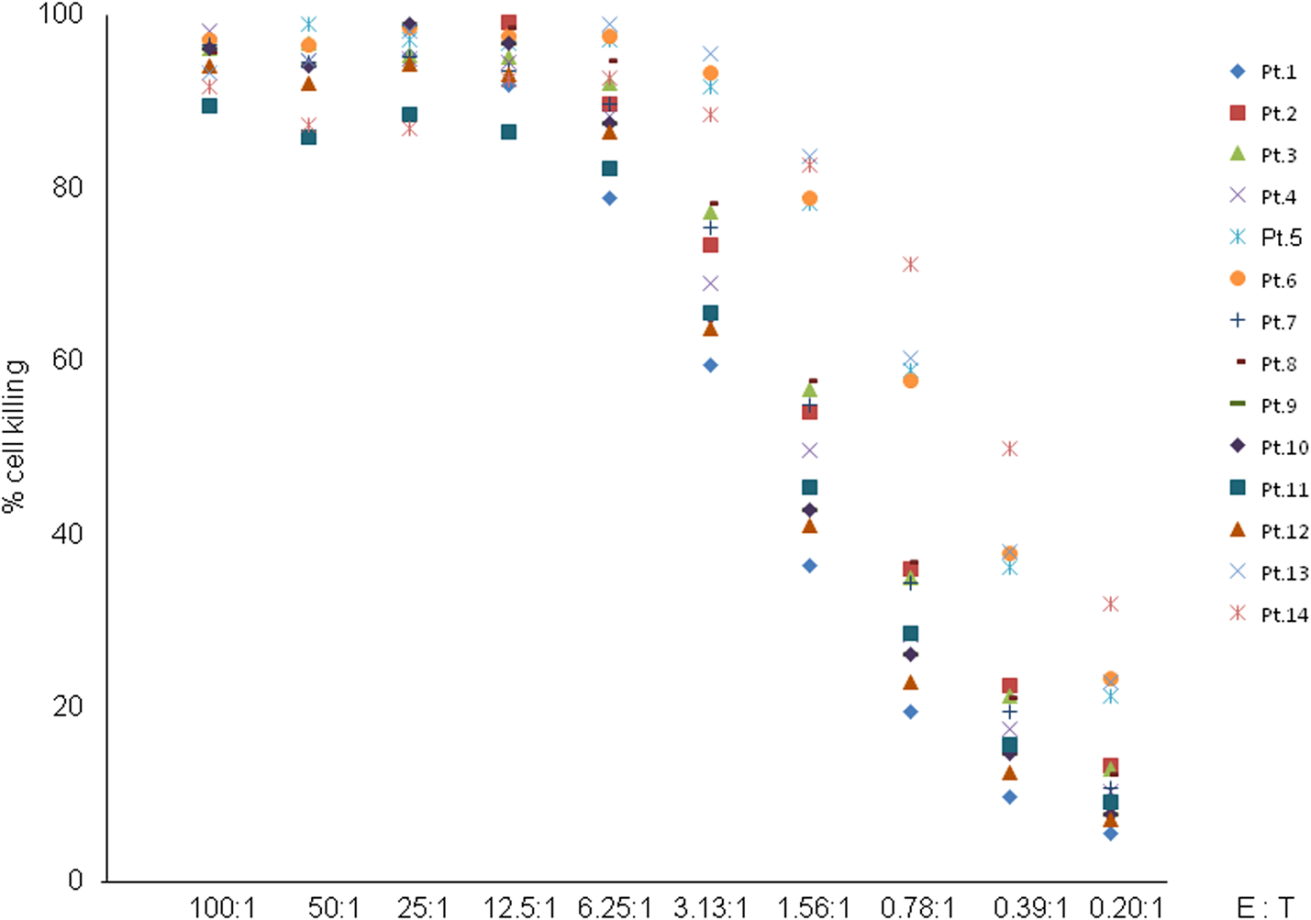
Cytotoxic activity of expanded NK cells against K-562 cells. 4 h cytotoxicity from 14 patients against K-562 target cells. Mean cell death in each patient at the indicated E:T ratios in triplicate cultures

We directly tested the cytotoxicity of each patient’s final product using the standard K-562 cells which is a NK-sensitive target. Expanded cells from all patients exerted strong cytotoxic activity against K-562 cells (Fig. 3). At a 6.25:1 effector to target cell ratio, 90.29 % of the K562 targets on average were killed by the expanded cells, and median EC50 value was 1.31 (range 0.39–2.61, Table 2). Representative data from patient no. 5, whose EC50 value was 0.63, appears in Additional file 3: Figure S2.

### Safety assessment

The overall toxicity in the 12 ITT patients who were administered NK cells more than twice, is summarized in Table 3. No patient showed toxicity that exceeded grade 3. We observed one patient with grade 3 lymphopenia (no. 3), one patient with grade 3 anorexia (no. 10) and one patient with grade 2 pleuritis (no. 8). These adverse events were caused by cancer progression. Grade 3 anorexia, fatigue, anemia, thrombocytopenia, and total bilirubin elevation were observed in one patient (no. 4). These adverse events were caused by bone marrow carcinomatosis and obstructive jaundice brought on by the progression of gastric cancer. Grade 3 neutropenia and anemia was observed in one patient (no. 11) administered with S-1. These hematological toxicities were transient and were alleviated by drug withdrawal. Grade 1 fever elevation and fatigue associated with NK cell infusion was observed in one patient. The frequency and severity of toxicity did not increase with dose escalation of infused cells, and there were no severe or unexpected toxicity related to the NK cell infusion. Thus, the maximum tolerated dose was not reached.

**Table 3:** Maximum toxicity per patient

Toxicity	Cohort 1 (n = 5)		Cohort 2 (n = 4)		Cohort 3 (n = 3)		All cohort (n = 12)	
	Grade		Grade		Grade		Grade	
	1–2	3	1–2	3	1–2	3	1–2	3
Hematological								
Neutropenia	0	0	1	0	0	1	1	1
Lymphopenia	3	1	3	0	0	0	6	1
Anemia	3	1	3	1	2	0	8	2
Thrombocytopenia	2	1	1	0	0	0	3	1
Elevated AST	2	0	1	0	0	0	3	0
Elevated ALT	0	0	1	0	0	0	1	0
Elevated total billirubin	0	1	2	0	1	0	3	1
Non-hematological								
Fatigue	2	0	3	0	3	0	8	0
Fever	1	0	0	0	0	0	1	0
Anorexia	0	1	0	1	0	0	0	2
Nausea	0	0	0	0	2	0	2	0
Diarrhea	0	0	2	0	0	0	2	0
Constipation	1	0	2	0	0	0	3	0

### Clinical efficacy

The evaluation of clinical outcome is shown in Table 4. No patient showed tumor shrinkage during the observation period up to 4 weeks after their last treatment. Of the 12 ITT patients, 5 (41.7 %) presented stable disease (SD) and 7 (58.3 %) presented progressive disease (PD). Of the 10 PP patients, 5 (50 %) presented SD and 5 (50 %) presented PD. Thus, in the PP analysis, the response rate and the disease control rate (DCR, complete response + partial response + SD) in this study were 0 and 50.0 %, respectively.

**Table 4:** Tumor response

No. of patients	Response				Response rate (%) (95 % CI)	Disease control rate (%) (95 % CI)
	CR	PR	SD	PD		
ITT population n = 12	0	0	5	7	0 (0)	41.7 (15.17–72.33)
PP population n = 10	0	0	5	5	0 (0)	50.0 (18.71–81.29)

*ITT* intention -to-treat, *PP* per protocol, *CR* complete response, *PR* Partial response, *SD* stable disease, *PD* progressive disease, *95* *%CI* 95 % confidence interval

### Immunological monitoring

**Fig. 4:**
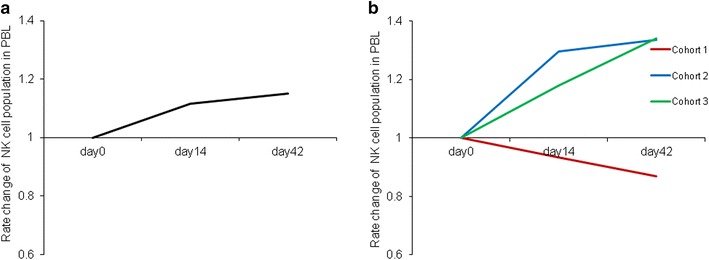
Longitudinal plots of NK cell population in PBLs plotted according to their deviation from the baseline. Mean levels in all patients (**a**) and levels in each cohort (**b**) are shown. *PBLs* peripheral blood lymphocytes

**Fig. 5:**
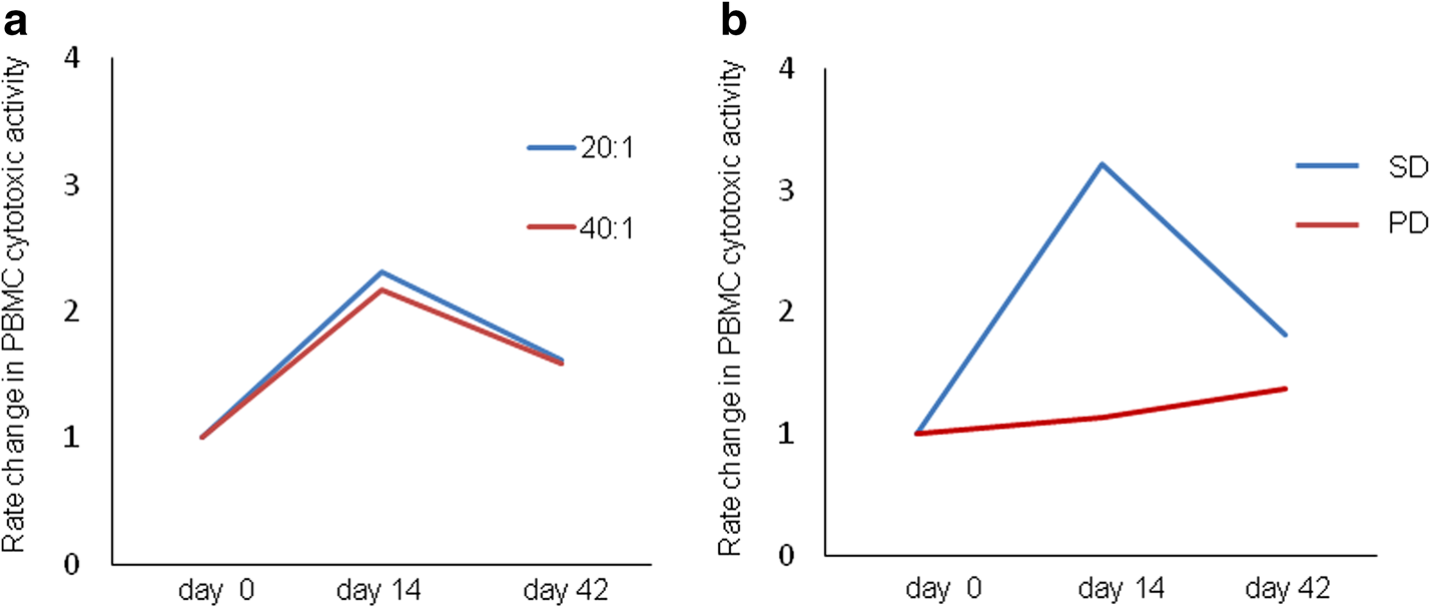
Longitudinal plots of cytotoxic activity of PBMCs against K-562 cells plotted according to the deviation from the baseline. Mean levels in all patients at indicated E:T ratios (**a** *blue* 20:1, *red* 40:1) and levels for the tumor responses at 40:1 E/T ratio (**b**) are shown. *PBMCs* peripheral blood mononuclear cells, *SD* stable disease, *PD* progressive disease

Immunological monitoring was performed for per protocol evaluable patients using their PBMCs before and after the NK cells infusion. As indicated in Fig. 4a and Additional file 4: Table S2, the average NK cell population in peripheral blood lymphocytes (PBLs) slightly increased until day 42 after NK cell infusion. Evaluated by cohort, the population of NK cells in cohort 2 and 3 increased after the infusion of NK cells, whereas there was a gradual decrease in cohort 1 (Fig. 4b). Next, we tested the cytotoxicity of PBMCs against K-562 targets and found that cytotoxicity increased in 80 % of patients after NK cell infusion (Additional file 5: Table S3). On day 14, average PBMC cytotoxic increased more than twice the day 0 baseline value; while it gradually decreased, it remained higher than day 0 until day 42 (Fig. 5a). This change in PBMC cytotoxic activity was similar across all cohorts and seemed to be independent of the number of cells that were infused. However, PBMC cytotoxic activity differed according to objective tumor response, and the average in patients with SD increased more than three times on day 14 and was much higher than in patients with PD (Fig. 5b). Cytokine serum levels did not change significantly after NK cell infusion (Table 5).

**Table 5:** Serum cytokines concentration before and after NK cell therapy

	Baseline (day 0) (pg/ml)	Post administration (day 42) (pg/ml)	p value
IL-1β	4.007 ± 7.155	6.438 ± 13.22	0.25
IL-1RA	893.3 ± 2154	983.0 ± 2550	1
IL-2	37.16 ± 22.54	37.45 ± 29.48	0.8203
IL-4	2.456 ± 0.6683	2.600 ± 0.7691	0.4961
IL-5	4.029 ± 2.191	4.171 ± 3.156	1
IL-6	38.62 ± 47.26	45.43 ± 58.69	0.3594
IL-7	3.827 ± 3.984	4.724 ± 6.224	0.8203
IL-8	26.94 ± 9.571	30.73 ± 16.71	0.4258
IL-9	31.33 ± 28.77	32.87 ± 37.69	0.4258
IL-10	26.28 ± 30.72	27.71 ± 39.37	0.3008
IL-12p70	37.28 ± 50.31	47.22 ± 83.46	0.9102
IL-13	14.87 ± 21.99	19.05 ± 36.70	0.8203
IL-15	44.33 ± 20.73	43.67 ± 28.17	0.8203
IL-17	199.2 ± 89.43	185.1 ± 109.2	0.4961
IFN-γ	92.13 ± 119.4	105.4 ± 141.8	0.3594
TNF-α	32.04 ± 45.28	38.69 ± 59.64	0.0977
bFGF	51.92 ± 31.51	53.38 ± 45.84	0.7344
PDGF-BB	172.2 ± 108.3	191.0 ± 219.0	0.9102
G-CSF	55.23 ± 26.89	58.08 ± 40.79	0.9102
GM-CSF	90.58 ± 75.85	94.29 ± 97.23	0.7344
IP-10	287.1 ± 40.88	389.2 ± 204.4	0.3008
MCP-1	133.6 ± 33.18	122.1 ± 52.29	0.2031
MIP-1α	4.972 ± 3.239	4.214 ± 2.073	0.1641
MIP-1β	275.0 ± 86.68	300.6 ± 154.0	1
Eotaxin	116.4 ± 193.8	123.2 ± 233.4	0.7344
RANTES	2157 ± 396.8	2445 ± 580.7	0.2031
VEGF	92.02 ± 30.85	113.2 ± 79.16	0.9102

Values are expressed as the mean ± SD. The paired t test was used to determine statistical differences

*NS* not significant

## Discussion

In this phase I clinical trial consisting of advanced digestive cancer patients, we explored the safety and feasibility of adoptive transfer of NK cells elicited by a novel expansion method using RN-T cells. We generated pure (median 90.96 %) NK cells on a large scale without prior purification. These cells expressed activating markers such as NKG2D and CD16 implicated in cytotoxicity and ADCC activity and exerted strong cytotoxic activity against K-562 cells in vitro. Not only did our proposed method produce fully functional NK cells in large numbers, the process was safe, well tolerated and highly reproducible.

NK cells occur in low numbers in PBMCs and obtaining the number necessary for adoptive transfer of NK cells is technically challenging. Unlike T cells, which readily respond to a variety of stimuli, it is generally difficult to expand NK cells from a small amount of blood from patients with advanced cancer who in general tend to have impaired immune function. There are few previous reports in which a NK cell expansion method produced pure cells in sufficient quantities from a small amount of blood without prior purification of the NK cells [26]. IL-2 can induce proliferative responses in human NK cells, but as suggested by existing reports, it can induce up to a 50-fold expansion after being cultured for 2 weeks [27–29]. Recently, Alici et al. reported that anti-CD3 and IL-2 induced 1600-fold NK expansions after 20 days from the blood of patients with myeloma [30]. However, the cytotoxicity of these cells against K562 cells was <10 % at 1:1 E/T ratio [30], which is much lower than the results achieved in this present in vitro study (average cytotoxicity 57.54 or 38.77 % at 1.56:1 or 0.75:1 E/T ratio).

We observed that chemokine receptors CXCR3, CXCR4 and CX3CR1 were relatively highly expressed in expanded NK cells, with a median in the ITT population, of 45.47, 37.71, 43.74 % respectively. These molecules are considered to be involved in the accumulation of intratumoral NK cells [31–34]. Thus, the NK cells elicited by our system are believed to have excellent properties as adoptive transferred cell in cancer immunotherapy. The percentage of CD16+ NK cells in the final product was relatively low compared to that observed in our previous study (average 55.4 vs 96.8 %) [25]. We observed by flow cytometer that CD16 expression on NK cells decreased approximately 30 % after being thawed and this may be attributed to us using thawed samples in the present study, whereas fresh samples were analyzed in our previous study.

Although no clinical responses were observed in 10 PP patients, the in vitro cytotoxicity of PBMCs against K-562 targets increased in 80 % of patients post adoptive NK cell transfer. The average cytotoxic activity on day 14 increased more than two times after NK cells infusion, and the increase continued until day 42 post-transfer. In their clinical trial for patients with metastatic melanoma or renal cell carcinoma, Parkhurst et al. documented the persistence, function and phenotype of autologous NK cells after adoptive transfer [35]. In their study, the peripheral blood lymphocyte 1 week post treatment consisted of an average of 83 % NK cells, which was much higher than in our clinical study (21 % at 2 weeks post-transfer). This difference in results probably stems from the higher number of transferred cells used in their clinical trial (47 × 10^9^ per dose) versus the current trial (0.5 or 1.0 or 2.0 × 10^9^ per dose). Additionally, their study involved lymph depleting chemotherapy and systemic IL-2 administration combined with NK cell transfer; these procedures were not done in our trial.

As previously mentioned, the focus of this clinical trial was to assess the safety of the NK cells produced with our expansion method. These cells had a relatively high level of functionality which made them promising however, we felt it was also important to know their level of toxicity before focusing on their efficacy. For this reason, although studies like those presented above have received better efficacy with combined treatment such as lymph depletion and any cytokine support, we chose a monotherapy where a relatively small number of NK cells were administered as the best option to assess the safety of the expanded cells.

To overcome the limited clinical efficacy of NK cell-based monotherapy, several strategies have been suggested including the combination of various monoclonal antibodies [26]. Within the setting of NK cell-based therapy, much attention has been given to the KIR-ligand mismatch phenomenon. Lack of KIR-HLA class I interactions has been associated with potent NK-mediated antitumor efficacy in acute myeloid leukemia patients upon haplo-identical stem cell transplantation [36] or NK cells infusion [6, 37] from KIR mismatched donors. Thus, a KIR ligand-mismatched donor is likely to provide the best chance for clinical response. Adoptive transfer of allogeneic NK cells from a KIR ligand-mismatched donor elicited by our novel method is expected to offer advantages over autologous NK cells. Since our method does not stimulate T cell proliferation, this could be an important clinical advantage because it avoids the risk of graft-versus-host disease in allogeneic NK cell therapy. Immune checkpoint blockade with antibodies to CTLA-4 and PD-1 represents a promising cancer therapy that aims to restore an efficient antitumoral response mediated by T cell [38]. As a corollary to targeting negative regulators of T cells, blocking inhibitory signals of NK cells with anti-KIR [39] or anti-Tim-3 [40] antibodies is also an attractive therapy to combine with NK cell therapy. CD16 expression of expanded NK cells produced in our study is relatively high. Therefore, adoptive transfer of these NK cells combined with tumor-specific monoclonal antibodies has the potential to trigger strong ADCC responses. This type of combined therapy with IgG1 monoclonal antibodies is likely to be promising. Having confirmed that our expanded cells are safe to administer, we are currently conducting clinical trials combining them with cetuximab for colorectal cancer and trastuzumab for gastric cancer patients in order to assess their efficacy. (The UMIN Clinical Trials Registry ID: UMIN000013378).

## Conclusion

We have shown that our novel NK expansion system using RN-T cells is effective at producing large numbers of fully functional NK cells that are pure. The data here also demonstrates that adoptive transfer of these NK cells is safe and very well tolerated by patients who had failed standard cancer therapy. NK cell transfer as a monotherapy is generally unsatisfactory and although no clinical responses were observed in patients, knowing that these cells can be safely administered will allow us to make an attempt at improving their efficacy by combining them with other reagents. We believe that these expanded cells have the potential to be efficacious in a combination treatment, since we observed that transferred NK cells persisted in the peripheral circulation of patients and exerted cytotoxicity in vitro. The NK cell expansion method suggested here could be applied to various NK cell-based therapies and can be combined with a range of treatment modalities. The advantage of our novel NK cell generation method can be summarized as follows: (1) large number of highly pure and functional cells (2) no need for purification including the use of a magnetic beads sorting system (3) improved safety through the use of autologous RN-T cells rather than transgenic or cancer cells as a stimulator (4) requires only a small amount of blood (5) requires low-serum culture conditions (0.5–1.0 %) and (6) high reproducibility. We believe that this expansion method is an innovative tool for NK cell-based cancer immunotherapy.

## Additional files

Additional file 1:
**Table S1.** Summary of NK cell expansion from 31 cancer patients (supplementary information of reference #25). Additional file 2:
**Figure S1.** Representative flow cytometry dot-plots for each population of expanded cells in patient no.14 (1st culture). Additional file 3:
**Figure S2.** Cytotoxic activity of the final product from patient 5 against K-562 cells. Mean cell death at the indicated E:T ratios in triplicate cultures. EC 50 is the value corresponding to the E:T ratio needed to reduce the cytotoxicity by 50 % from maximum lysis. Additional file 4:
**Table S2.** Change in NK population in PBL. Additional file 5:
**Table S3.** Change in cytotoxic activity of PBMCs against K-562 cells during and after NK cell therapy.
