# Supplementary figures and images for: Phase I clinical trial of autologous NK cell therapy using novel expansion method in patients with advanced digestive cancer
Source: J Transl Med. 2015 Aug 25;13:277. doi: 10.1186/s12967-015-0632-8 (PMC4548900; doi:10.1186/s12967-015-0632-8)

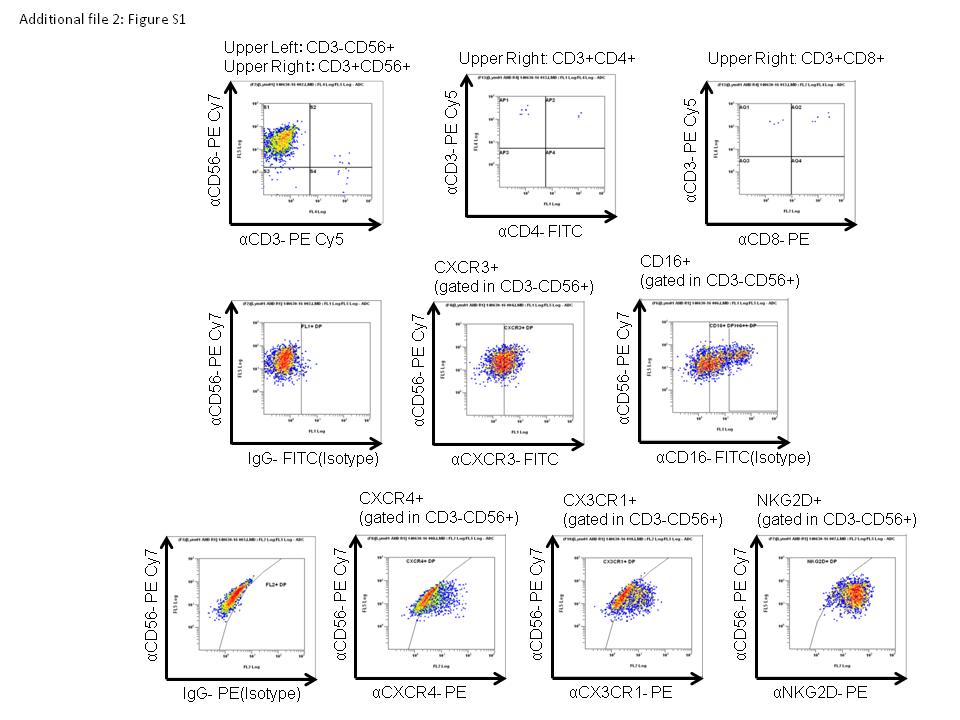

Supplement: Additional file 2: — Figure S1. Representative flow cytometry dot-plots for each population of expanded cells in patient no.14 (1st culture). [file 12967_2015_632_MOESM2_ESM.tif]

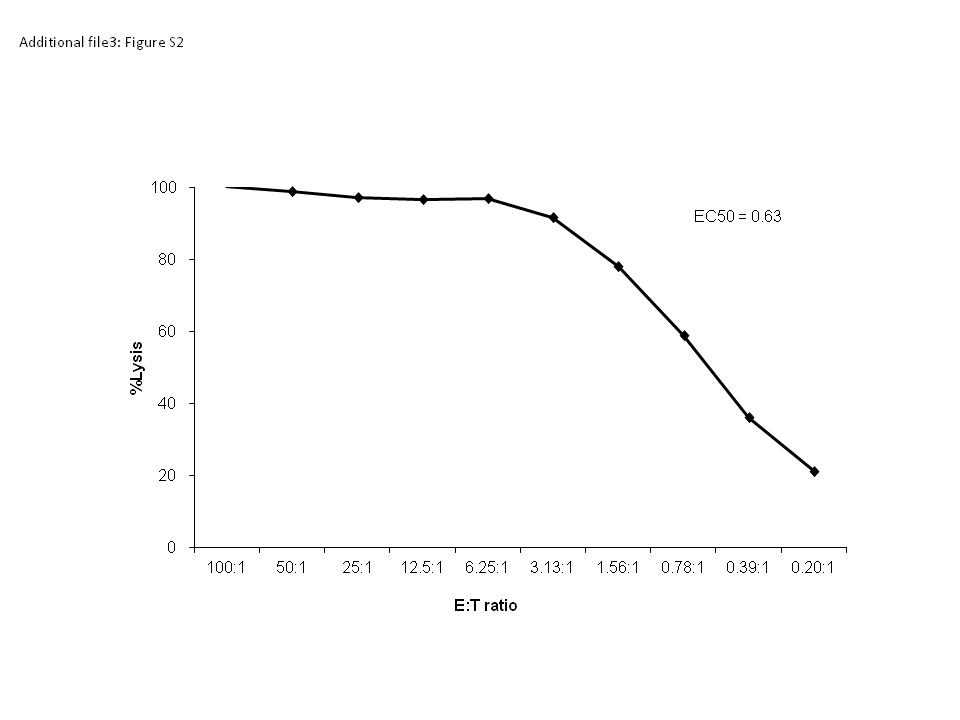

Supplement: Additional file 3: — Figure S2. Cytotoxic activity of the final product from patient 5 against K-562 cells. Mean cell death at the indicated E:T ratios in triplicate cultures. EC 50 is the value corresponding to the E:T ratio needed to reduce the cytotoxicity by 50 % from maximum lysis. [file 12967_2015_632_MOESM3_ESM.tif]
